# Safety and efficacy of the MNK/VEGFR inhibitor JDB153 combined with the anti-PD-1 antibody serplulimab in patients with advanced pancreatic cancer refractory to standard treatment: a single-arm, phase Ib/II study protocol

**DOI:** 10.3389/fphar.2026.1805590

**Published:** 2026-06-15

**Authors:** Xiaorui Zhu, Hao Wei, Dan Cao, Huanji Xu

**Affiliations:** 1 Division of Abdominal Tumor Multimodality Treatment, Cancer Center, West China Hospital, Sichuan University, Chengdu, China; 2 Division of Abdominal Tumor, Department of Medical Oncology, Cancer Center, Laboratory of Abdominal Tumor Immunology and Microenvironment, State Key Laboratory of Biotherapy, West China Hospital, Sichuan University, Chengdu, China

**Keywords:** clinical trial protocol, immunotherapy, JDB153, mnk inhibitor, pancreatic cancer, serplulimab, VEGFR inhibitor

## Abstract

**Introduction:**

Pancreatic cancer remains a leading cause of death with poor prognosis. Current standard chemotherapies offer limited survival benefits, and no standard treatment exists for patients failing two lines of therapy. Preclinical evidence suggests that targeting MNK and VEGFR pathways can remodel the immunosuppressive tumor microenvironment and enhance immunotherapy efficacy. This study evaluates the safety and efficacy of JDB153 (an MNK/VEGFR inhibitor) combined with serplulimab (an anti-PD-L1 antibody) in refractory advanced pancreatic cancer.

**Methods and analysis:**

This is a prospective, open-label, single-center, phase Ib/II clinical trial (NCT07175389). It will enroll patients with advanced pancreatic adenocarcinoma who have progressed after standard therapies. The study follows a Simon’s two-stage design. The initial phase will enroll 10 patients to test safety and preliminary efficacy. Patients will receive oral JDB153 and intravenous serplulimab. If at least one patient responds, the study will expand to a total of 32 patients. The primary endpoints are safety and ORR. Secondary endpoints include DFS, OS, and PFS. Safety will be graded using NCI-CTCAE version 5.0. Comprehensive biomarker analyses (PD-L1 expression, spatial immune profiling, next-generation sequencing, et al.) are integrated.

**Discussion:**

Patients with advanced pancreatic cancer need effective options that are less toxic than chemotherapy. Immunotherapy alone often fails in this disease due to the immunosuppressive microenvironment. By combining MNK/VEGFR inhibition with PD-1 blockade, this study investigates a promising strategy to overcome immune resistance and establish a novel treatment paradigm for advanced pancreatic adenocarcinoma.

## 1 Introduction

Pancreatic cancer remains a formidable global health challenge, ranking as the sixth leading cause of cancer-related mortality worldwide (Bray et al., 2024). Characterized by an insidious onset and rapid progression, approximately 80% of patients are diagnosed at an advanced stage (Li et al., 2004; Ryan et al., 2014), resulting in a 5-year survival rate of merely 13% (Siegel et al., 2025).

Current first-line treatments for advanced pancreatic cancer predominantly rely on chemotherapy regimens, specifically FOLFIRINOX (irinotecan, 5-fluorouracil/leucovorin, and oxaliplatin), GnP (gemcitabine plus nab-paclitaxel), and NALIRIFOX (liposomal irinotecan, 5-fluorouracil/leucovorin and oxaliplatin) (National Comprehensive Cancer Network, 2025). The PRODIGE 4/ACCORD 11 trial demonstrated a median overall survival (OS) of 11.1 months with FOLFIRINOX, significantly outperforming gemcitabine monotherapy (Conroy et al., 2011). Similarly, the MPACT study showed that the GnP regimen improved OS to 8.5 months (Von Hoff et al., 2013). Despite these advances, the median progression-free survival (PFS) remained disappointing, at roughly 6 months, and over half of patients experienced disease progression or death within 1 year (Conroy et al., 2011; Von Hoff et al., 2013). Even the recent NAPOLI-3 study indicated that while NALIRIFOX improved OS compared to GnP (11.1 months vs. 9.2 months), the PFS was still limited to 7.4 months (Wainberg et al., 2023).

The situation for second-line treatment is also difficult and generally follows a cross-over chemotherapy principle (National Comprehensive Cancer Network, 2025). While studies have confirmed the efficacy of 5-fluorouracil-based regimens after gemcitabine failure and the benefit of gemcitabine-based regimens after 5-fluorouracil failure, the median OS in the second-line setting rarely exceeds 7 months (Wang-Gillam et al., 2016; Oettle et al., 2014; De La Fouchardière et al., 2024). Crucially, there is no established standard care for third-line treatment, necessitating the development of novel and effective subsequent therapies (National Comprehensive Cancer Network, 2025).

Pancreatic cancer is characterized by abnormal blood vessel formation and high interstitial pressure, creating a barrier that prevents immune cells from entering the tumor (Li et al., 2019). Targeting the vascular endothelial growth factor receptor (VEGFR) can normalize these blood vessels and remodel the immunosuppressive microenvironment, thereby facilitating cytotoxic T cells infiltration and enhancing antitumor immunity (Goel et al., 2011; Liu et al., 2022). However, no antiangiogenic agents or immunotherapy have been approved for pancreatic cancer to date. This unmet need underscores the urgency of identifying novel therapeutic targets and combination strategies to improve outcomes in this refractory disease.

The regulation of protein translation plays a vital role in gene expression, and eIF4E is a core component of this process (Sonenberg and Hinnebusch, 2009; Chen et al., 2023). EIF4E initiates protein synthesis by binding the mRNA 5′-cap to form the eIF4F complex (Sonenberg and Hinnebusch, 2009; Chen et al., 2023). Its activity is strictly regulated by the Ras/Raf/MEK/ERK/MNK and PI3K/Akt/mTOR pathways, particularly through phosphorylation at Ser209 b y MNK kinases (Fabbri et al., 2021; Jin et al., 2021; Fernandez et al., 2023). EIF4E is often overexpressed in pancreatic cancer, and drives the translation of oncogenic proteins that are related to tumor growth, metastasis, drug resistance, and poor prognosis (Ma et al., 2019; Adesso et al., 2013; Fang et al., 2025; Li et al., 2024). Preclinical research shows that eFT508, a MNK kinase inhibitor, significantly reduces the proliferation and survival of pancreatic cancer cell lines by inducing apoptosis (Li et al., 2024). Blocking MNK can inhibit tumor growth without affecting normal cells, making it a safe therapeutic target (Jin et al., 2021; Li et al., 2024).

**FIGURE 1:**
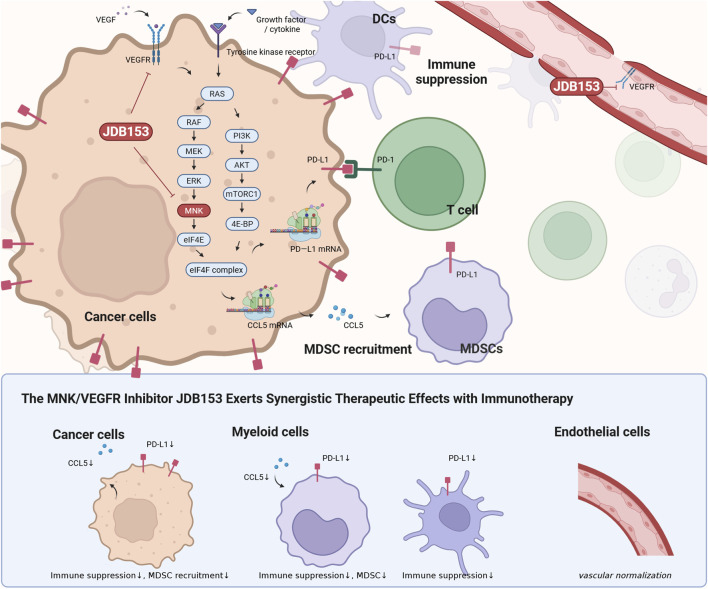
Mechanism of the dual MNK/VEGFR inhibitor JDB153 synergizing with anti-PD-1 therapy in pancreatic cancer. This diagram illustrates the synergistic therapeutic mechanism of JDB153 and serplulimab. Centrally, the MNK1/2-eIF4E axis integrates oncogenic signals from the MAPK and PI3K pathways to selectively drive the translation of pro-tumorigenic mRNAs, including PD-L1 and CCL5. By inhibiting MNK, JDB153 directly downregulates PD-L1 protein expression on tumor cells and immunosuppressive myeloid cells (MDSCs/DCs), thereby lowering the threshold for T cell activation. Simultaneously, its VEGFR inhibition facilitates vascular normalization, reducing interstitial fluid pressure and alleviating the physical barriers characteristic of the pancreatic cancer microenvironment. This dual action reshapes the cold tumor microenvironment into a hot one, characterized by significantly increased infiltration and cytotoxic function of CD8^+^ T cells. Combined with the anti-PD-1 antibody serplulimab, this strategy overcomes immunotherapy resistance, offering a potent approach to enhance anti-tumor immunity in advanced pancreatic adenocarcinoma. Abbreviations: CCL5, C-C motif chemokine ligand 5; DCs, dendritic cells; MDSCs, myeloid-derived suppressor cells; MNK, mitogen-activated protein kinase-interacting kinase; PD-1, programmed cell death protein 1; PD-L1, programmed death-ligand 1; VEGF, vascular endothelial growth factor; VEGFR, vascular endothelial growth factor receptor.

Furthermore, MNK inhibition reconfigures the immune microenvironment and enhances immunotherapy through multiple mechanisms (Figure 1) (Bartish et al., 2023). Preclinical studies across various malignancies demonstrates that inhibiting MNK-mediated eIF4E phosphorylation selectively decreases PD-L1 protein expression on tumor cells, dendritic cells, and myeloid-derived suppressor cells (MDSCs) (Xu et al., 2019; Cerezo et al., 2018; Huang et al., 2021; Guo et al., 2021). Additionally, blocking the MNK1/2-eIF4E axis impairs the production of pro-inflammatory factors like CCL5, and reduces the recruitment of immunosuppressive myeloid cells while increasing the infiltration and cytotoxic activity of CD8^+^ T cells (Xu et al., 2019; Huang et al., 2021; Guo et al., 2021).

JDB153 is a novel dual inhibitor targeting both MNK and VEGFR. In lung cancer models, JDB153 has demonstrated potent antitumor efficacy by inhibiting cell proliferation and angiogenesis, and reshapes the tumor microenvironment by increasing the infiltration of CD8^+^ T cells (Xu et al., 2025). Immune checkpoint inhibitors (ICIs) have shown safety but limited efficacy in pancreatic cancer (Marabelle et al., 2020; Renouf et al., 2022; Fu et al., 2023; Cheng et al., 2024; Wainberg et al., 2020; Kamath et al., 2020). Although no clinical study has yet investigated the efficacy of JDB153 in combination with immunotherapy, we hypothesize that JDB153 can synergize with serplulimab, an anti-PD-1 antibody, to enhance the immune response against pancreatic adenocarcinoma, thereby improving the overall therapeutic efficacy. This synergistic effect is anticipated to overcome immunotherapy resistance mechanisms, offering a novel and potentially more effective treatment strategy for advanced pancreatic adenocarcinoma. This study aims to evaluate the safety and efficacy of JDB153 combined with serplulimab in patients with advanced pancreatic cancer who have failed standard therapies.

## 2 Methods and analysis

**FIGURE 2:**
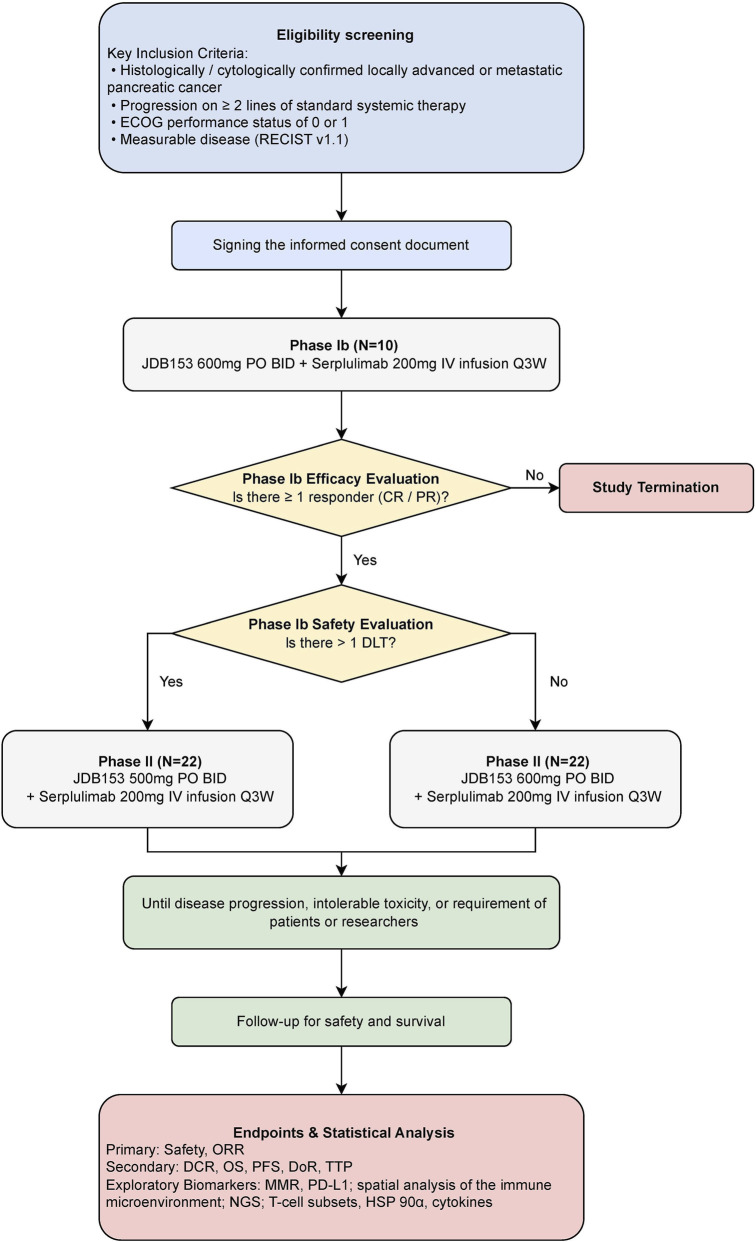
Study Design and Participant Flow Abbreviations: BID, twice daily; CR, complete response; DCR, disease control rate; DLT, dose-limiting toxicity; DoR, duration of response; ECOG, Eastern Cooperative Oncology Group; HSP90α, heat shock protein 90 alpha; IV, intravenous; MMR, mismatch repair; N, number of patients; NGS, next-generation sequencing; ORR, objective response rate; OS, overall survival; PD-L1, programmed death-ligand 1; PFS, progression-free survival; PO, orally; PR, partial response; Q3W, every 3 weeks; RECIST, Response Evaluation Criteria in Solid Tumors; TTP, time to progression.

This study is a prospective, open-label, single-center, phase Ib/II clinical trial, and will be conducted at the West China Hospital, Sichuan University. The trial has received approval from the Ethics Review Committee of West China Hospital, Sichuan University, and is registered with ClinicalTrials.gov (NCT07175389). The reporting of this protocol follows the Standard Protocol Items: Recommendations for Interventional Trials (SPIRIT) guidelines (Figure 2).

### 2.1 Study objectives

The primary objective of this study is to evaluate the safety and efficacy of JDB153 tablets combined with serplulimab injection in patients with advanced pancreatic cancer who have experienced disease progression following standard treatments.

The primary endpoints focus on safety profile and objective response. Safety is assessed based on adverse events (AEs) graded via the National Cancer Institute Common Terminology Criteria for Adverse Events (NCI-CTCAE) version 5.0, including the incidence of dose-limiting toxicities (DLTs), serious adverse events (SAEs), and treatment-related adverse events (TRAEs). Efficacy is primarily measured by the objective response rate (ORR), defined as the proportion of patients achieving complete response (CR) or partial response (PR) according to Response Evaluation Criteria in Solid Tumors (RECIST) version 1.1.

Secondary endpoints include the disease control rate (DCR), as well as OS, PFS, duration of response (DoR), and time to progression (TTP). DCR is defined as proportion of patients achieving CR, PR, and stable disease (SD). OS is defined as the time from study enrollment to death from any cause. PFS is defined as the time from study enrollment to the first documented disease progression or death. DoR is defined as the time from first documented objective response (CR or PR) to disease progression or death, and TTP is defined as the time from study enrollment to disease progression.

### 2.2 Study design

The study employs a Simon’s two-stage design, which permits early termination for futility and minimizes patient exposure to an ineffective regimen. The initial phase (phase Ib) will enroll 10 participants to evaluate the safety and preliminary efficacy of the combination. Patients will receive JDB153 at a dose of 600 mg orally twice daily (BID) and serplulimab at a dose of 200 mg via intravenous infusion every 3 weeks. If no patient achieves an objective response, the study will be terminated. The study will also be terminated in the event of unresolved SAEs or death. If one or more patient achieves, the trial will proceed to phase II. This phase will enroll additional patients to reach a total sample size of 32. If more than one patient in the phase Ib experiences a DLT, the dose of JDB153 for the phase II cohort will be reduced to 500 mg orally BID. Otherwise, the dose will remain at 600 mg orally BID. If DLT occur in one or more patients at the 500 mg BID dose level, the study will be terminated. If four or more responses are observed among the 29 evaluable patients, the null hypothesis will be rejected, indicating that the regimen merits further investigation.

Treatment will continue until documented disease progression (PD), intolerable AEs, withdrawal of consent by the patient, or a maximum duration of 24 months.

### 2.3 Eligibility criteria

The study population consists of patients with histologically or cytologically confirmed locally advanced or metastatic pancreatic adenocarcinoma and must have experienced disease progression after at least one line of standard systemic therapy (Table 1). Additional key inclusion criteria require patients to be between 18 and 75 years of age, have a life expectancy of at least 12 weeks, and maintain an Eastern Cooperative Oncology Group (ECOG) performance status of 0 or 1. Patients must possess at least one measurable lesion according to RECIST version 1.1.

**TABLE 1:** Inclusion and exclusion criteria for patient selection.

Item	Criteria description
Inclusion criteria	
Diagnosis	Histologically or cytologically confirmed locally advanced unresectable or metastatic pancreatic adenocarcinoma
Demographics	Age between 18 and 75 years, male or female
Life expectancy	Life expectancy of at least 12 weeks
Performance status	ECOG performance status of 0 or 1
Prior therapy	Disease progression following standard systemic therapy. Disease progression occurring during or within 6 months of completing adjuvant chemotherapy is considered as failure of one line of systemic therapy
Measurable disease	Presence of at least one measurable lesion according to RECIST v1.1. Longest diameter ≥10 mm if measured by spiral CT or ≥20 mm if measured by conventional CT or MRI.
Organ function	Adequate bone marrow and organ function, defined by laboratory results obtained within 7 days prior to enrollment. Patients must not have received blood transfusion, granulocyte colony-stimulating factor, or other medical supportive treatment within 14 days prior to the first dose of study drug • Hemoglobin: ≥90 g/L • Platelets: ≥75 × 10^9/L • White blood cells: ≥3.0 × 10^9/L • Absolute neutrophil count: ≥1.5 × 10^9/L • Total bilirubin: ≤1.5× ULN (≤3× ULN if liver metastases are present) • ALT and AST: ≤2.5× ULN (≤5× ULN if liver metastases are present) • Creatinine: ≤1.5× ULN.
Informed consent	Voluntary participation with a signed and dated written informed consent form
Exclusion criteria	
Prior malignancy	History of other malignant tumors with disease free survival <5 years. Exceptions include cured basal cell carcinoma of the skin, cured cervical carcinoma *in situ*, and gastrointestinal tumors cured via endoscopic mucosal resection
Prior immunotherapy	Prior treatment with anti-PD-1, or anti-PD-L1 inhibitors
Immunodeficiency	Diagnosis of immunodeficiency diseases or HIV infection
Active infection	Severe uncontrolled acute infection, defined as infection causing a fever >38 °C
Viral hepatitis	Active hepatitis B or hepatitis C infection, defined as: HBV DNA ≥2000 IU/mL (or 1 × 10^4 copies/mL), or HCV RNA ≥ lower limit of detection
Organ dysfunction	Severe hepatic or renal insufficiency
Tuberculosis	Active tuberculosis infection within 1 year prior to the first dose. Patients with a history of active tuberculosis >1 year prior may be eligible if there is no current evidence of active infection as determined by the investigator
Lung disease	History of interstitial lung disease or non-infectious pneumonitis that is symptomatic or may interfere with the assessment of pulmonary toxicity
Cardiac history	History of myocardial infarction within 3 months prior to the first dose
Gastrointestinal disorders	History of chronic diarrhea or presence of complete intestinal obstruction
Autoimmune disease	Active autoimmune disease or a documented history of autoimmune disease with a risk of recurrence (e.g., requiring immunosuppressive therapy after organ transplantation). Exceptions allowed type 1 diabetes mellitus, hypothyroidism requiring hormone replacement only, and skin diseases not requiring systemic treatment (e.g., vitiligo, psoriasis, alopecia)
Corticosteroid use	Current use of systemic corticosteroids (>10 mg/day prednisone equivalent) or other immunosuppressive medications within 14 days prior to the first dose. Exceptions allowed inhaled or topical steroids; adrenal replacement (≤10 mg/day prednisone equivalent); short-term (≤7 days) prophylaxis (e.g., contrast dye allergy) or treatment of non-autoimmune conditions (e.g., delayed-type hypersensitivity)
Severe comorbidities	Presence of other severe acute or chronic medical or psychiatric conditions that may affect organ function, making the patient unsuitable for this trial according to the investigator’s judgment
Concurrent trials	Participation in another clinical trial with an investigational drug within 4 weeks prior to the first dose
Pregnancy and lactation	Women who are pregnant or breastfeeding, or patients of childbearing potential (men or women who have been post-menopausal for less than 1 year) unwilling to use effective contraception methods
Hypersensitivity	History of allergic or hypersensitivity reactions to any study drug components
Investigator judgment	Patients deemed unsuitable for participation in this clinical trial by the investigator

Abbreviations: ALT, alanine aminotransferase; AST, aspartate aminotransferase; CT, computed tomography; ECOG, eastern cooperative oncology group; HBV, hepatitis B virus; HCV, hepatitis C virus; HIV, human immunodeficiency virus; IU, international units; MRI, magnetic resonance imaging; PD-1, programmed cell death protein 1; PD-L1, programmed death-ligand 1; RECIST, response evaluation criteria in solid tumors; ULN, upper limit of normal.

Candidates will be excluded if they have a history of other malignancies within the past 5 years, uncontrolled concurrent illnesses, or significant autoimmune or immunodeficiency diseases.

### 2.4 Recruitment and screening

Screening procedures will be conducted after obtaining informed consent (Table 2). These include the collection of demographic data, comprehensive medical history, a full physical examination encompassing height, weight, and vital signs, and ECOG performance status. Laboratory assessments will cover complete blood count, urinalysis, blood chemistry, coagulation, tumor markers, cardiac enzymes, thyroid function, and serology for hepatitis B virus (HBV), hepatitis C virus (HCV), and human immunodeficiency virus (HIV). Patients with positive HBsAg must undergo an HBV DNA test, and those with positive HCV antibodies requires an HCV RNA test. Women of childbearing potential must undergo a serum β-HCG test. Cardiac safety will also be assessed via electrocardiograms, and echocardiography. Tumor imaging will be performed using contrast-enhanced computed tomography (CT) or magnetic resonance imaging (MRI) of the abdomen, pelvis, and chest. Quality of life (QoL) scores will be recorded at baseline according to EORTC QLQ-C30 or QLQ-BIL21.

**TABLE 2:** Schedule of patient visits and assessments.

Study period	Screening		Treatment		Follow-up	
Visit window *a*	Within 2 weeks prior to C1D1	Within 1 week prior to C1D1	Within 1 week prior to C2-xD1	Every 8 weeks ( ± 1 week)	4 weeks ( ± 1 week) after EOT	Every 12 weeks ( ± 1 week) after EOT
Clinical assessments						
Demographics	✓	​	​	​	​	​
Medical history	✓	​	​	​	​	​
Physical examination *b*	​	✓	✓	​	✓	​
ECOG performance status	​	✓	✓	​	✓	​
Quality of life	​	✓	​	✓	✓	​
Adverse events	​	​	✓	​	✓	​
Concomitant medications	✓	​	✓	​	✓	​
Electrocardiograms	✓	​	✓	​	✓	​
Echocardiogram	✓	​	​	​	✓	​
Laboratory assessments						
Complete blood count	​	✓	✓	​	✓	​
Urinalysis	​	✓	✓	​	✓	​
Blood chemistry test	​	✓	✓	​	✓	​
Coagulation test	​	✓	✓	​	✓	​
HBV, HCV, and HIV test *c*	✓	​	​	​	✓	​
Tumor markers test	​	✓	✓	​	✓	​
Cardiac enzymes test	​	✓	✓	​	✓	​
Thyroid function test	​	✓	✓	​	✓	​
Pregnancy test *d*	​	✓	​	​	✓	​
Tumor assessment						
CT/MRI imaging *e*	✓	​	​	✓	✓	​
Biomarker exploration						
Tumor sample	​	✓	​	​	​	​
Blood sample	​	✓	✓	​	​	​
Follow-up						
Survival status	​	​	​	​	​	✓
Subsequent therapy	​	​	​	​	​	✓

Abbreviations: C1D1, cycle 1 day 1; C2-x, cycle two and subsequent cycles; CT, computed tomography; ECOG, eastern cooperative oncology group; EOT, end of treatment; HBV, hepatitis B virus; HCG, human chorionic gonadotropin; HCV, hepatitis C virus; HIV, human immunodeficiency virus; MRI, magnetic resonance imaging.

Footnotes.

a Cycle Definition: Each treatment cycle is 3 weeks. Assessments listed under the Treatment column are to be performed prior to drug administration on day 1 of cycle two and subsequent cycles. Cycle 1 day 1 assessments are covered under Screening.

b Physical Examination: Includes vital signs (blood pressure, heart rate, respiratory rate, temperature), height, and weight.

c HBV, HCV, and HIV, Test: Includes HBsAg, HBsAb, HBeAg, HBeAb, HBcAb, HCV, antibody, and HIV, antibody screening. Patients with positive HBsAg must undergo further quantitative HBV DNA, testing. Patients with positive HCV, antibody must undergo further quantitative HCV RNA, testing.

d Pregnancy Test: Only applicable to women of childbearing potential. A serum β-HCG, test must be performed within 7 days prior to the first dose and at the safety follow-up visit.

e Tumor Imaging: Contrast-enhanced CT, or MRI, of the chest, abdomen, and pelvis. Scans performed during Screening serve as the baseline. Subsequent scans are performed every 8 weeks (±7 days), until disease progression or withdrawal of consent. The same imaging modality should be used throughout the study.

### 2.5 Data collection and assessments

Tumor response will be evaluated every 8 weeks according to RECIST version 1.1. Given the mechanism of immunotherapy, pseudoprogression is a possibility. If disease progression is suspected radiologically but the patient is clinically stable, treatment may continue, and a confirmatory scan will be performed at least 4 weeks later, utilizing irRECIST criteria.

Safety will be assessed continuously throughout the study. Adverse events will be graded according to NCI-CTCAE version 5.0. Assessments will include physical examinations, ECOG performance status, and comprehensive laboratory panels (complete blood count, urinalysis, blood biochemistry, coagulation, tumor markers, cardiac enzymes, and thyroid function) performed within 1 week prior to the start of each new cycle. QoL scores will be collected every 8 weeks.

As the single-agent pharmacokinetic profile of JDB153 has been established in a prior phase I study (unpublished data), sparse sampling in cycle one combined with trough concentration monitoring in subsequent cycles is sufficient to assess potential drug-drug interactions while minimizing patient burden. Blood samples will be collected pre-dose, and at 2, 4, and 8 h post-dose in cycle 1 only.

Biomarker exploration is a key component of this trial. Patients will consent to the use of archival tumor tissue previously obtained and blood samples collected within 1 week prior to treatment. Analyses will include PD-L1 expression, mismatch repair (MMR) status, single-cell spatial proteomics and transcriptomics, and next-generation sequencing (NGS). PD-L1 is evaluated as a predictive marker for immunotherapy response and is mechanistically linked to JDB153 activity via MNK-eIF4E-mediated translational regulation. Peripheral blood markers will include T-cell subsets, heat shock protein 90α, and cytokines (IL-6, IL-8, IL-17, CRP) to capture dynamic immune changes and identify potential predictive biomarkers for treatment response.

### 2.6 Interventions and dose modifications

Differentiation between targeted therapy toxicity and immune-related adverse events will be guided by timing of onset, histologic findings when available, and response to corticosteroids. Management of adverse reactions follows a stepwise approach based on severity. Appropriate supportive care will be administered, such as antidiarrheals for diarrhea, antiemetics for nausea, and hepatoprotective agents for liver dysfunction.

Dose adjustments for JDB153 are based on toxicity grades. If a ≥ grade 3 TRAE occurs, the JDB153 dose may be reduced to 500 mg BID. If a ≥ grade 3 TRAE recurs, a further reduction to 400 mg BID is permitted. More than two dose reductions and dose re-escalation after a reduction are not permitted. A new treatment cycle may begin only when neutrophils ≥1.5 × 10^9/L, platelets ≥75 × 10^9/L, and non-hematologic toxicities have recovered to ≤ grade 1. If treatment is interrupted for more than 2 weeks due to hematological toxicity, JDB153 should be permanently discontinued.

Dose reductions for serplulimab are not permitted. If toxicity results in a treatment delay of more than 12 weeks for any grade ≥2 adverse event that does not recover to grade ≤1, serplulimab will be permanently discontinued (Table 3).

**TABLE 3:** Treatment adjustment for serplulimab-related adverse events.

Immune-related adverse event	Severity	Treatment adjustment
Severe cutaneous adverse reaction	Suspected SJS, TEN, or DRESS	Suspend use
	Confirmed SJS, TEN, or DRESS	Permanently discontinue
Colitis	Grade 2	Suspend use *a*
	Grade 3 or 4	Permanently discontinue
Hepatitis	ALT or AST: >3 to ≤5× ULN (>3 to ≤5× baseline if baseline is abnormal); orTotal bilirubin: >1.5 to ≤3× ULN (>1.5 to ≤3× baseline if baseline is abnormal)	Suspend use *a*
	ALT or AST: >5× ULN (>5× baseline if baseline is abnormal); orTotal bilirubin: >3× ULN (>3× baseline if baseline is abnormal)	Permanently discontinue
Pneumonitis	Grade 2	Suspend use *a*
	Grade 3 or 4	Permanently discontinue
Endocrine toxicity *b*	Grade 3 or 4	Suspend use until clinically stable, or permanently discontinue for severe cases
Nephritis	Grade 2 or 3 creatinine increased	Suspend use *a*
	Grade 4 creatinine increased	Permanently discontinue
Nervous system toxicity	Grade 2	Suspend use *a*
	Grade 3 or 4	Permanently discontinue
Myocarditis	Grade 2, 3, or 4	Permanently discontinue
Ocular toxicity	≥ Grade 2 toxicity not resolved to grade 1 within 2 weeks with local treatment, or requiring systemic treatment	Permanently discontinue
Infusion-related reaction	Grade 1 or 2	Interrupt infusion or reduce infusion rate
	Grade 3 or 4	Permanently discontinue
Other irAEs	Grade 2 or 3 (based on type and severity)	Suspend use *a*
	Grade 3 or 4 encephalitis; orGrade 3 or 4 Guillain−Barre syndrome; orGrade 4 or recurrent grade 3	Permanently discontinue

Abbreviations: ALT, alanine aminotransferase; AST, aspartate aminotransferase; DRESS, drug reaction with eosinophilia and systemic symptoms; irAE, immune-related adverse event; SJS, Stevens-Johnson syndrome; TEN, toxic epidermal necrolysis; ULN, upper limit of normal.

Footnotes.

a Treatment may resume when the adverse event resolves to grade 0–1 and the daily corticosteroid dose has been tapered to ≤10 mg prednisone (or equivalent). If the adverse event does not resolve to grade 0–1, or if the corticosteroid dose cannot be reduced to ≤10 mg/day prednisone equivalent within 12 weeks, serplulimab must be permanently discontinued.

b Depending on clinical severity, treatment may be withheld for grade 2 endocrine toxicities until symptoms improve with hormone replacement therapy. Treatment may resume once acute symptoms have resolved.

### 2.7 Statistical analysis

The sample size is calculated based on a Simon’s two-stage optimum design. The null hypothesis (P0) assumes a historical ORR of 5% for second-line treatment, while the alternative hypothesis (P1) anticipates an ORR of 20%. With a one-sided alpha of 0.05 and a beta of 0.2, the first-stage enrollment is 10 patients (n1 = 10), with a futility boundary of 0 responders (r1 = 0). The total planned enrollment is 29 patients (n = 29), with a rejection boundary of three responders (r = 3). Accounting for a 10% dropout rate, the total enrollment target is 32 patients.

Analyses will follow the intention-to-treat principle. The full analysis set and per-protocol set will be used for efficacy, while the safety analysis set will cover all patients who received at least one dose of the study drug.

Continuous variables will be presented as mean ± standard deviation or median with range, and categorical variables as frequencies and percentages. ORR and DCR will be reported with 95% Clopper-Pearson confidence intervals. Time-to-event endpoints (OS, PFS, DoR, TTP) will be estimated using the Kaplan-Meier method. Univariate and multivariable Cox proportional hazards models will be used to explore the impact of baseline characteristics and biomarkers on survival outcomes. Multivariable Cox regression models will include prior treatment characteristics as covariates to adjust for potential heterogeneity in baseline therapies. All statistical tests will be two-sided with a significance level of 0.05, performed using SPSS version 22.0 or higher.

## 3 Discussion

Current clinical practice offers no established standard of care for patients with pancreatic cancer who progress after two lines of systemic therapy (National Comprehensive Cancer Network, 2025). These patients typically present with poor performance status and high symptom burden, limiting their ability to tolerate further intensive cytotoxic regimens. There is an urgent need for effective strategies that extend survival without compromising quality of life. This study aims to evaluate the safety and efficacy of a chemotherapy-free strategy using JDB153 combined with serplulimab.

ICIs have revolutionized the treatment of most solid tumors, such as melanoma and lung cancer (Topalian et al., 2015; Wu, 2022). However, they have historically failed to demonstrate meaningful efficacy in pancreatic cancer. This failure is primarily due to the “cold” nature of the pancreatic tumor microenvironment, characterized by a dense stroma and few immune cells (Ullman et al., 2022; Fan et al., 2020). Current guidelines recommend immunotherapy mainly for patients with microsatellite instability-high (MSI-H) or mismatch repair deficiency (dMMR) (National Comprehensive Cancer Network, 2025), but this group represents only about 1.0%–2.5% of patients (Grant et al., 2021; Ahmad-Nielsen et al., 2020; Luchini et al., 2021). Even within this biomarker-selected population, the efficacy in pancreatic cancer is modest. The KEYNOTE-158 trial reported an ORR of 18.2% and a median OS of 4.0 months (Marabelle et al., 2020). Previous trials attempting to combine immunotherapy with standard chemotherapy have also produced disappointing results (Renouf et al., 2022; Fu et al., 2023; Cheng et al., 2024; Wainberg et al., 2020; Kamath et al., 2020). Therefore, new strategies are needed. Remodeling the tumor microenvironment through combination therapies to relieve immunosuppression may be key to increasing sensitivity to immunotherapy (Zhang et al., 2022). For example, a phase II study combined the HDAC inhibitor entinostat with nivolumab in advanced pancreatic cancer. Among 27 evaluable patients, three achieved PR, with a median DoR of 10.2 months. Although the primary endpoint was not met, it showed that modifying the tumor microenvironment can help overcome resistance (Baretti et al., 2024). Similarly, a phase I study of the FAK inhibitor defactinib combined with pembrolizumab and gemcitabine showed a DCR of 80% and a median OS of 8.3 months in refractory pancreatic cancer (Wang-Gillam et al., 2022).

MNK is the only kinase that can directly regulate eIF4E activity (Buxade et al., 2008). However, MNK inhibitors alone have a limited ability to suppress eIF4E phosphorylation and tumor growth, and require combination with other agents (Li et al., 2023). Clinical data supports that targeting the MNK pathway can reprogram the immune landscape and make tumors more responsive to ICIs. A phase II study of the MNK inhibitor eFT508 combined with PD-1 blockade showed the regimen was well-tolerated and achieved partial responses in heavily pretreated patients. The most common grade 3 or 4 TRAEs were increased liver enzymes and rash, and about 18% of patients discontinued treatment due to AEs (El-Khoueiry et al., 2020). Another phase II study showed that eFT508 combined with avelumab had acceptable safety and preliminary activity in colorectal cancer. Common AEs included diarrhea, constipation, fatigue, myalgia or arthralgia, hypercalcemia and rash (Hubbard et al., 2019). Additionally, preliminary data from the KICKSTART trial in non-small cell lung cancer suggested MNK inhibition might enhance pembrolizumab activity. Although the OS data was immature and the PFS results did not meet statistical significance, there was a signal of efficacy. However, grade 3 or 4 AEs were more common in the combination arm compared to the placebo arm (67% vs. 37%) (Flaherty, 2024).

Combining anti-angiogenic agents with ICIs can promote the maturation of antigen-presenting cells and the activation of T cells. It also reduces the number of suppressive cells in the tumor (Kudo, 2020). Recent studies in pancreatic cancer have validated this approach. The NASCA trial evaluated surufatinib (a VEGFR inhibitor) combined with an anti-PD-1 antibody and chemotherapy. It reported a significantly improved ORR of 51.1% compared to chemotherapy alone. The most common grade 3 or higher TRAEs was decreased neutrophil count (Jia et al., 2025). Similarly, other trials have combined anti-angiogenic agents like anlotinib or bevacizumab with PD-1 inhibitors have shown promising response rates and survival benefits (Sha et al., 2024; Ying et al., 2025). These results confirm that vascular normalization can synergize with immunotherapy to enhance the anti-tumor immune response in pancreatic adenocarcinoma.

JDB153 is a unique dual inhibitor that targets both MNK and VEGFR, simultaneously suppressing the translation of oncogenic proteins and inducing tumor vascular normalization. Preclinical models in non-small cell lung cancer have demonstrated that JDB153 exhibits anti-tumor activity and synergizes with anti-PD-1 therapy (Xu et al., 2025). Our team previously conducted a phase II study of serplulimab combined with GnP chemotherapy and radiotherapy. That study confirmed that serplulimab is well tolerated in advanced pancreatic cancer (Cheng et al., 2025). We hypothesize that JDB153 will create a favorable immune environment that allows serplulimab to work effectively.

A major strength of this study is its chemotherapy-free design. Patients in the third-line setting are often frail and cannot tolerate intensive chemotherapy regimens. JDB153 and serplulimab are expected to have a more manageable toxicity profile compared to traditional chemotherapy. Safety is a primary concern and we have implemented a rigorous safety monitoring plan. The study utilizes a Simon’s two-stage design. This allows us to assess preliminary efficacy in a small cohort before expanding enrollment. This protects patients from exposure to an ineffective regimen. The study also includes a comprehensive biomarker analysis. We will analyze tumor tissue and blood samples to investigate changes in the immune microenvironment and translational machinery. Understanding these biological changes will help us identify which patients are most likely to benefit from this combination. It may also reveal mechanisms of resistance that could inform future research.

There are limitations of this study. As a single-arm, phase Ib/II study, the sample size is relatively small. The lack of a randomized control arm limits our ability to definitively compare efficacy against other potential palliative treatments. However, given the lack of standard care in this setting, comparing results against historical controls is a valid approach for early-phase investigation. If the primary endpoints are met, larger randomized clinical trials will be necessary to confirm these findings.

In conclusion, this study explores a novel therapeutic combination for a patient population with significant unmet needs. By integrating MNK inhibition, VEGFR inhibition, and PD-1 blockade, we aim to overcome the immunosuppressive barriers of pancreatic cancer. This research will provide valuable evidence regarding the clinical application of combining translational and angiogenic inhibitors with immunotherapy. Ultimately, we hope to establish a new, effective, and well-tolerated treatment option for patients with advanced pancreatic cancer.

## Data Availability

The original contributions presented in the study are included in the article/supplementary material, further inquiries can be directed to the corresponding authors.
